# Infrared Thermography with High Accuracy in a Neonatal Incubator

**DOI:** 10.1007/s10439-022-02937-w

**Published:** 2022-03-02

**Authors:** Keisuke Hamada, Eiji Hirakawa, Hidetsugu Asano, Hayato Hayashi, Takashi Mine, Tatsuki Ichikawa, Yasuhiro Nagata

**Affiliations:** 1 Department of Clinical Engineering, Nagasaki Harbor Medical Center, Nagasaki, Nagasaki Japan; 2 Department of Comprehensive Community Care Systems, Nagasaki University Graduate School of Biomedical Sciences, Nagasaki, Nagasaki Japan; 3 Department of Neonatology, Kagoshima City Hospital, Kagoshima, Kagoshima Japan; 4 Research & Development Group, Technical Department, Atom Medical Corporation, Saitama, Saitama Japan; 5 Department of Clinical Oncology, Nagasaki Harbor Medical Center, Nagasaki, Nagasaki Japan; 6 Department of Gastroenterology, Nagasaki Harbor Medical Center, Nagasaki, Nagasaki Japan; 7 Department of Community Medicine, Nagasaki University School of Biomedical Sciences, Nagasaki, Nagasaki Japan; 8 Leading Medical Research Core Unit, Nagasaki University Graduate School of Biomedical Sciences, Nagasaki, Nagasaki Japan

**Keywords:** IRT, Premature infants, Calibration, Non-invasive, Monitoring, Thermoregulation

## Abstract

As the accuracy of body temperature measurement is especially critical in premature infants on admission to the neonatal intensive care unit (NICU), noninvasive measurement using infrared thermography (IRT) has not been widely adopted in the NICU due to a lack of evidence regarding its accuracy. We have established a new calibration method for IRT in an incubator, and evaluated its accuracy and reliability at different incubator settings using a variable-temperature blackbody furnace. This method improved the accuracy and reliability of IRT with an increase in percentage of data with mean absolute error (MAE) < 0.3 °C to 93.1% compared to 4.2% using the standard method. Two of three IRTs had MAE < 0.1 °C under all conditions examined. This method provided high accuracy not only for measurements at specific times but also for continuous monitoring. It will also contribute to avoiding the risk of neonates' skin trouble caused by attaching a thermistor. This study will facilitate the development of novel means of administering neonatal body temperature.

**Supplementary Information:**

The online version contains supplementary material available at 10.1007/s10439-022-02937-w.

## Introduction

Body temperature is an important health indicator in the formulation of a clinical management plan, and appropriate management of body temperature is required for maintenance of normal physiological functions and to facilitate prompt recovery after illness.^28, 38^ Especially stringent body temperature management is required for premature infants, and it has been known since the 1960s that reducing heat loss in the first few days of life can improve the survival rate of premature infants.^36^ Newborn infants lose heat at a rate two to three times higher than adults, and without treatment, neonatal body temperature usually decreases by 0.1–0.3°C per minute.^4, 41^

The importance of controlling body temperature in clinical practice has been widely acknowledged since the mercury thermometer was first used for medical purposes in the late 18th century.^42^ Since the 1980s, predictive thermometers have commonly been used to measure temperature in newborn infants. Moreover, as neonates require strict body temperature management, reliable and sustainable measurements are necessary using a thermistor probe. However, the insufficiently keratinized epidermal barrier of premature skin makes the attachment of sensors to the body difficult, resulting in inaccurate body temperature measurements. Therefore, there have been a number of clinical and laboratory trials to examine various noninvasive methods for measurement of body temperature. The infrared thermometer is already used as a noninvasive means of measuring body temperature, not only in daily clinical practice but also for non-medical purposes at home.^30^ Infrared thermography (IRT) is a similar method that can be used to determine the temperature of an object by measuring infrared emissions. As IRT has a wide field of view (FOV), it can simultaneously measure multiple objects, and is therefore useful for screening at airports.^9, 13^ For example, IRT has been used for fever screening in the coronavirus disease 2019 (COVID-19) pandemic.^5, 8^ However, IRT must be used in combination with a Food and Drug Administration (FDA)-approved thermometer due to its low level of accuracy.^11^

IRT was first applied in medical research at Middlesex Hospital in London and the Royal National Hospital for Rheumatic Diseases in Bath, both in the UK, from 1959 to 1961.^32^ In addition, IRT has been used to analyze changes in body temperature due to exercise.^15, 18, 43^ The medical application of IRT in neonates was first examined in the 1970s. Pomerance *et al.*^29^ reported that the skin directly above highly vascular organs, such as the heart, liver, and kidneys, has a higher body surface temperature compared to other areas. Abbas *et al*.^1^ reported that respiratory rate can be monitored by IRT using a method based on the difference between inspiratory and expiratory nostril temperature. Knobel *et al.*^22^ reported that abdominal temperature is lower in patients with neonatal necrotizing enterocolitis (NEC) compared to in those without evidence of this condition. Furthermore, both abdominal and leg temperatures of neonates at gestational age 23–28 weeks measured by IRT over the first week after birth were reported to be similar to those measured using a thermistor-based contact method.^23^

For use in clinical practice, methods for measurement of body temperature require a high degree of accuracy due to the critical importance of body temperature management. This is especially true in newborn infants. However, IRT generally has insufficient accuracy for medical use, and it is therefore necessary to improve the accuracy of neonatal infrared thermography (NIRT) to measure the body temperature of newborn infants. Abbas *et al.*^2^ suggested that different temperature compensation equations should be used according to the measurement environment, i.e., convective neonatal incubators, kangaroo mother care, and open radiant warmers.

There have been no previous reports of IRT installed inside the incubator, with previous studies involving making a hole in the hood or measuring temperature from the outside. These studies simply compared the skin surface temperature measured by the infrared camera and thermistor. It has a problem that they did not compare at the same body area due to the thermistor generating heat itself. Furthermore, there have been no previous reports about the emissivity of neonates' skin and how it changes with growth. Hence, this study focused on examining and improving the accuracy of measurement of the object by IRT installed in the neonatal incubator before assessment of neonates’ body temperature and emissivity. We confirmed that our method had general applicability by examining three commercially available thermal cameras, i.e., FLIR A35 defined as IRT-1 and 2, and FLIR Lepton 3.5 defined as IRT-3.

### Conventional Correction Equation and Error Factors of IRT

As it is dangerous to use a heating element, such as a blackbody furnace, inside a neonatal incubator, we used a non-heating blackbody (BB_rs_) as a reference source in this study. A blackbody furnace (BB_obj_) was used as a substitute for the neonate and was not used as a reference source.

**Figure 1:**
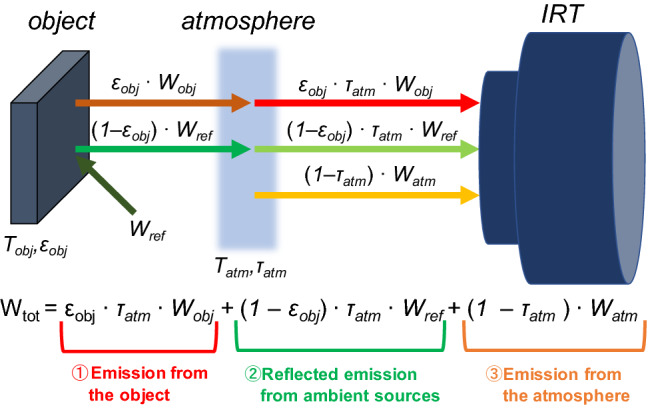
Total emission from the object.

When the measurement object is an opaque body, IRT receives the sum of emissions from the object, reflected emissions from ambient sources, and emissions from the atmosphere (Fig. 1).^16, 26, 27, 39, 40^

1 $$W_{{{\text{tot}}}} = \varepsilon_{{{\text{obj}}}} \cdot \tau_{{{\text{atm}}}} \cdot W_{{{\text{obj}}}} + \left( {{1} - \varepsilon_{{{\text{obj}}}} } \right) \cdot \tau_{{{\text{atm}}}} \cdot W_{{{\text{ref}}}} + \, \left( {{1} - \tau_{{{\text{atm}}}} } \right) \cdot W_{{{\text{atm}}}}$$

where *ε*_obj_ is the emissivity of the object, *τ*_atm_ is the transmittance of the atmosphere, *W*_obj_ is the emission of the object, *W*_ref_ is the reflected emission of ambient sources, and *W*_atm_ is the emission of the atmosphere.

Subsequent equation expansion is performed by radiance. Temperature conversion and radiance conversion are performed using the Stefan–Boltzmann equation (2).

2 $$\begin{aligned} W & = \sigma \times T^{4} \\ T & = \sqrt[4]{{\frac{W}{\sigma }}} \\ \end{aligned}$$

where *σ* is the Stefan–Boltzmann constant (equal to 5.670367 × 10^−^^8^W/m^2^·K^4^).

When IRT is used inside the convective neonatal incubator, the emission of the ambient sources (*W*_amb_) is equal to the emission of the atmosphere (*W*_atm_) due to the uniform temperature in the incubator.^20^ Therefore, the emission from the object detected by IRT (*W*_det1_) can be obtained from Eq. (3):

3 $$W_{{{\text{obj}}}} = \frac{{W_{{{\text{det1}}}} - W_{{{\text{amb}}}} }}{{\varepsilon_{{{\text{obj}}}} \tau_{{{\text{atm}}}} }} + W_{{{\text{amb}}}}$$

Here, the transparency of the atmosphere (*τ*_atm_) is calculated from Eqs. (4) and (5). The equations used by the FLIR tools are as follows^17, 39^:

4 $$\tau \left( {d,\omega } \right) = K_{{{\text{atm}}}} \exp \left[ { - \sqrt d \left( {\alpha_{1} + \beta_{1} \sqrt \omega } \right)} \right] + \left( {1 - K_{{{\text{atm}}}} } \right)\exp \left[ { - \sqrt d \left( {\alpha_{2} + \beta_{2} \sqrt \omega } \right)} \right]$$

5 $$\omega \left( {\omega_{\% } ,T_{{{\text{atm}}}} } \right) = \omega_{\% } \cdot \exp \left( {h_{1} + h_{2} \cdot T_{{{\text{atm}}}} + h_{3} \cdot T_{{{\text{atm}}}}^{2} + h_{4} \cdot T_{{{\text{atm}}}}^{3} } \right)$$

where *τ* is the transmittance of the atmosphere, *K*_atm_ is the scaling factor for atmospheric damping (1.9), *d* is the distance from the IRT, *α*_1_ and *α*_2_ are attenuation factors for the atmosphere without water vapor, *β*_1_ and *β*_2_ are attenuation factors for water vapor, *ω* is the coefficient indicating the content of water vapor in the atmosphere, *ω*_%_ is the relative humidity, and *h*_1_ = 1.5587, *h*_2_ = 6.939×10^−2^, *h*_3_ = − 2.7816×10^−4^, and *h*_4_ = 6.8455 × 10^−7^.

Convective incubators with high temperature and humidity are used in the clinical management of premature infants, and both humidity and ambient temperature are known to affect the measurement of temperature by IRT.^3^ Even conventional IRT software can adjust for these parameters. The error factors of IRT can be divided into two types, i.e., external error factors that are due to the measurement environment and internal error factors that are due to the structure of the IRT device.^16^ The conventional equation (3) is generally used to correct the measurements. However, Eq. (3) takes external error factors into consideration but does not consider the influence of internal error factors. A cooled infrared thermograph cannot be used for IRT inside an incubator as the refrigerant could adversely affect the condition of the neonate. Furthermore, despite their advantages in terms of size and cost, the accuracy of uncooled IRTs is known to be inadequate.^31, 34^ Therefore, the use of an uncooled IRT requires the incorporation of internal errors, including emissions from the body of the IRT and the temperature characteristics of the sensor, into body temperature calculations.^16^ To cope with such errors associated with emissions from the body of the IRT that manifest as thermal drift of the focal plane array (FPA),^10, 24^ an uncooled IRT calibrates the temperature using a mechanical shutter during non-uniformity correction (NUC). To achieve high accuracy, calibration is required every time the incubator settings are changed to deal with errors associated with the temperature characteristics of the sensor. However, we found that IRT yields inaccurate measurements even with appropriate adjustment of all of the software parameters because NUC operation is intermittent. Standard two-point calibration cannot completely eliminate the influences of thermal drift as they fluctuate over time. In this study, a reference source was used based on the concept that both the measured object and a reference source would be equally influenced by thermal drift. Curcio and Haberman^14^ reported that IRT cannot accurately recognize temperature differences, and many groups have used an independent thermal reference system to improve the accuracy of measurements.^12^ A screening thermograph consists of an IRT and an external temperature reference source (ETRS).^19, 21^ Simpson *et al.*^37^ reported that the National Physical Laboratory (NPL) developed a multi-fixed-point source as an in-image calibration system. However, this system was not used in the present study because it was difficult to use inside the incubator. Therefore, a blackbody was used as a reference source the temperature of which was affected by the incubator settings, but it was not harmful when used inside the incubator due to its passive thermal control.

### Establishment of a Comparison Equation

**Figure 2:**
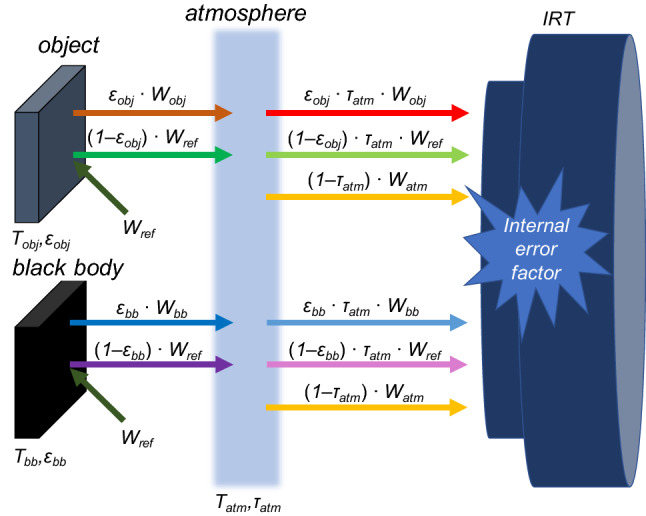
Total emissions from the object (BB_obj_) and the reference source (BB_rs_).

The emission from the blackbody furnace (BB_obj_) as a measured object and the blackbody (BB_rs_) as a reference source were measured simultaneously (Fig. 2). The estimated emission of BB_rs_ (*W*_rs_) was obtained from equation (6):

6 $$W_{{{\text{bb}}}} = \frac{{W_{{{\text{det2}}}} - W_{{{\text{amb}}}} }}{{\varepsilon_{{{\text{rs}}}} \cdot \tau_{{{\text{atm}}}} }} + W_{{{\text{amb}}}}$$

where *ε*_rs_ is the emissivity of BB_rs_ and *W*_det2_ is the emission from BB_rs_ detected by the IRT.

A comparison equation was established as correction equation (7) calculated from the internal emission of BB_rs_ measured by the thermistor (*W*_TMT-rs_) and the difference between equations (3) and (6):

7 $$W_{{{\text{COR1}}}} = W_{{{\text{obj}}}} - W_{{{\text{rs}}}} + W_{{{\text{TMT}} - {\text{rs}}}} = \frac{{W_{{{\text{det}}1}} - W_{{{\text{amb}}}} }}{{\varepsilon_{{{\text{obj}}}} \cdot \tau_{{{\text{atm}}}} }} - \frac{{W_{{{\text{det}}2}} - W_{{{\text{amb}}}} }}{{\varepsilon_{{{\text{rs}}}} \cdot \tau_{{{\text{atm}}}} }} + W_{{{\text{TMT}} - {\text{rs}}}}$$

where *W*_TMT-rs_ is the emission of the blackbody measured by the thermistor.

In a steady state, the emission from BB_rs_ (*W*_det2_) equilibrates with the emission of the ambient sources inside the incubator (*W*_amb_). Therefore, *W*_amb_ can be expressed as *W*_det2_:

8 $$W_{{{\text{COR}}1}} = \frac{{W_{{{\text{det}}1}} - W_{{{\text{det}}2}} }}{{\varepsilon_{{{\text{obj}}}} \cdot \tau_{{{\text{atm}}}} }} - \frac{{W_{{{\text{det}}2}} - W_{{{\text{det}}2}} }}{{\varepsilon_{{{\text{rs}}}} \cdot \tau_{{{\text{atm}}}} }} + W_{{{\text{TMT}} - {\text{rs}}}} = \frac{{W_{{{\text{det}}1}} - W_{{{\text{det}}2}} }}{{\varepsilon_{{{\text{obj}}}} \cdot \tau_{{{\text{atm}}}} }} + W_{{{\text{TMT}} - {\text{rs}}}}$$

When using a single-detector scanning camera, all points have identical parameters as they are all measured by the same detector.^25^ These values also include internal error, so not only external error but also internal error can be taken into consideration when using Eq. (8).

### Establishment of a Linear Equation

Mean absolute error (MAE) is defined as the mean of the difference in absolute values between the value calculated by each correction equation and the real temperature of the blackbody furnace (BB_obj_).

**Figure 3:**
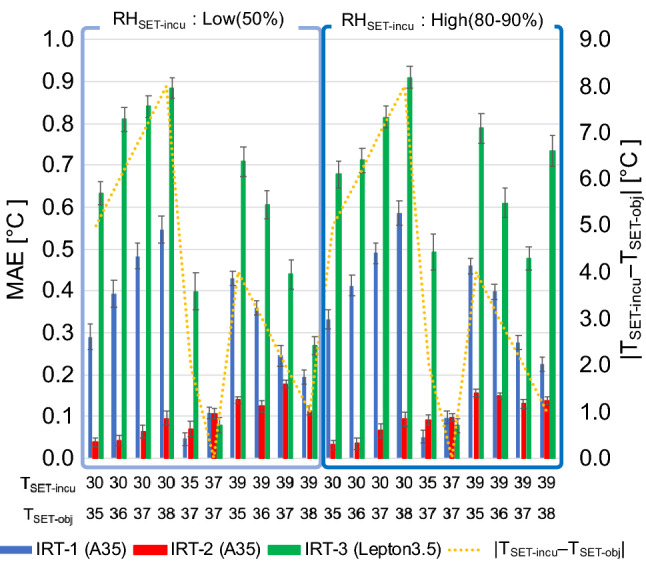
Differences in MAE under each set of conditions when corrected by Eq. (8). MAE of *T*_COR1_ are shown for each condition and each camera. The error bars show the standard deviation. The yellow dashed line indicates the absolute value of difference between *T*_SET-obj_ and *T*_SET-incu_. The conditions were divided into two groups according to the relative humidity (Low: 50%; High: 80%–90%).

When using Eq. (8), the MAE tended to increase when differences between the temperature inside the incubator and the temperature of BB_obj_ were large. Moreover, these tendencies differed between individual cameras. Furthermore, relative humidity inside the incubator had no influence on MAE (Fig. 3). Therefore, we postulated that the measurement accuracy would be improved by using regression analysis:

9 $$W_{COR2} = a \cdot \left( {\frac{{W_{{{\text{det}}1}} - W_{{{\text{det}}2}} }}{{\varepsilon_{{{\text{obj}}}} \cdot \tau_{{{\text{atm}}}} }}} \right) + W_{{{\text{TMT}} - {\text{rs}}}} + b$$

where *a* and *b* are the regression coefficients.

Regression analysis was performed using Eq. (9) and the data of Appendix Table S1–S3 and the values of the parameters were as follows: (IRT-1) *a*: 0.924892, *b*: 0.0704166, *R*^2^: 0.999931; (IRT-2) *a*: 0.9805749, *b*: 0.0821071, *R*^2^: 0.99997; (IRT-3) *a*: 1.1669497, *b*: −0.075513, and *R*^2^: 0.998043.

Moreover, while equation (9) showed good performance with regression analysis using 24 conditions, equation (10) showed good performance with the number of conditions reduced to two selected where the difference between the object to be measured (*T*_SET-obj_) and the incubator temperature (*T*_SET-incu_) was large: *T*_SET-incu_: 30°C, *T*_SET-obj_: 38°C; and *T*_SET-incu_: 39°C, *T*_SET-obj_: 35°C):

10 $$W^{\prime}_{{{\text{COR2}}}} = a^{\prime} \cdot \left( {\frac{{W_{{{\text{det}}1}} - W_{{{\text{det}}2}} }}{{\varepsilon_{{{\text{obj}}}} \cdot \tau_{{{\text{atm}}}} }}} \right) + W_{{{\text{TMT}} - {\text{rs}}}} + b^{\prime}$$

where *a′* and *b′* are the regression coefficients. The values of the parameters were as follows: (IRT-1) *a′*: 0.9267647, *b′*: 0.0649386; (IRT-2) *a′*: 0.9815677, *b′*: 0.0555107; (IRT-3) *a′*: 1.1510959, *b′*: −0.161881.

## Materials and Methods

**Figure 4:**
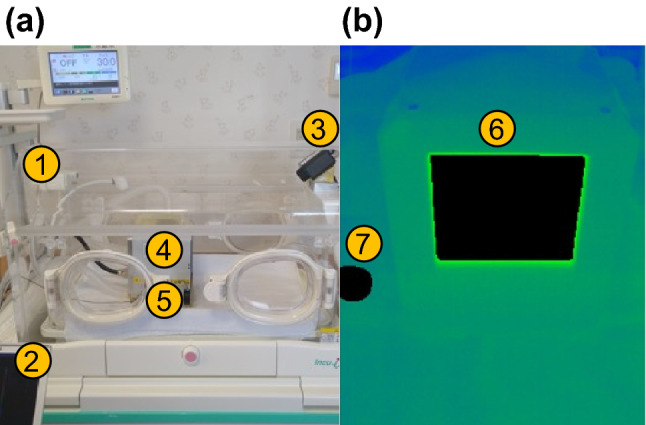
Incubator settings and thermal images of the IRT accuracy experiment. (a) IRT imaging inside the incubator: (1) convective neonatal incubator; (2) analysis workstation; (3) infrared thermography camera; (4) blackbody furnace (*ε* = 0.97); (5) blackbody (*ε* = 0.95). (b) IRT image of the blackbody furnace and the blackbody: (6) trace area of the blackbody furnace; (7) trace area of the blackbody.

The IRT was installed inside a convective neonatal incubator (Incu i; Atom Medical Corporation, Tokyo, Japan) and the temperature of the blackbody furnace (BB_obj_) was determined as the measured object (Fig. 4). The technical specifications of the blackbody furnace are listed in Table 1.

**Table 1:** Technical data of blackbody furnace

Product name	Caliber [inches]	Temperature resolution [°C]	Uniformity [°C]	Accuracy [°C]	Stability [°C]	Emissivity
SR800N-4A	4 × 4	0.001	± 0.01	± 0.015@ΔT < 0, ± 0.007@0 < T < 50, ± 0.015@ΔT > 50	± 0.003 @ΔT < ± 10	0.97 ± 0.02

The uncooled microbolometers IRT-1 and IRT-2 (A35; FLIR Systems Inc., Portland, OR, USA) and IRT-3 (Lepton 3.5; FLIR Systems Inc.) were used in this study. The technical specifications of the IRTs are listed in Table 2.

**Table 2:** Technical data of IRT

Product name	Spectral range [μm]	Operating range[°C]	Thermal sensitivity/NETD	Image sensor dimensions (pixels)	Detector pitch (*μ*m)	Accuracy
A35	7.5–13	− 25 ± 100	< 0.05 °C@30 °C /50 mK	320 × 256	17	± 5 °C or ± 5%
Lepton3.5	8–14	−10 ± 140	< 0.05 °C@30 °C /50 mK	160 × 120	12	± 5 °C or ± 5%

A35 with two cameras (IRT-1, IRT-2) was used to examine the differences within the same model, and a smaller power-saving model, Lepton 3.5 (IRT-3), was also used to compare the differences between machines. FLIR tools were used to analyze the results obtained with A35, while an analysis tool developed in house was used to analyze the results obtained with Lepton 3.5. In this study, the emissivity of the object was set to 1.0 to obtain the total emission from the object. A standard two-point calibration was not performed beforehand.

All exhaust gas from BB_obj_ was emitted to the outside of the incubator through the circuit so that the temperature and humidity inside the incubator were unaffected by the gas. As a reference source, a blackbody (BB_rs_) 3.8 cm in diameter and 1.5 cm in height, wrapped with tape of emissivity 0.95, was installed inside the same FOV. The thermistor (19X18-01; Nikkiso-Therm Co., Ltd., Tokyo, Japan) was placed inside the hole at the center of BB_rs_ to measure its internal temperature and was connected to a data logger (N543; Nikkiso-Therm Co., Ltd.). BB_obj_ and BB_rs_ were installed at the same distances from the IRT. To minimize the reflected radiation from the surroundings, the mattress was covered with a low-reflectance cloth commonly used for neonates. Relative humidity (*RH*_SET-incu_) and temperature inside the incubator (*T*_SET-incu_) were measured using the sensor of the incubator and were maintained at 30–39°C and 50%–90%, respectively. BB-1 temperatures (*T*_SET-obj_) were set to 35–38°C, which are widely accepted as within normal limits for neonates. The experiments were conducted under 24 different settings corresponding to common settings used for incubator management to examine their effects on measurement accuracy.

After confirming the stability of the temperature inside BB_rs_ under each condition, temperature data of the IRT were captured for 20 minutes under each condition. The data were obtained at a rate of once per second yielding a total of 1200 data points. The temperatures of BB_obj_ and BB_rs_ were obtained simultaneously from each image (Fig. 1b). Then, the top 10% upper and lower values were omitted and the average values for each image were calculated. Similarly, the average values were calculated for each condition.

All data were analyzed using JMP^®^ Pro (Ver. 16.0.0; SAS Institute Inc., Cary, NC, USA). MAE was calculated each second under each condition and the average values were compared. When calculating MAE, the setting temperature of BB_obj_ was used as the real temperature because the furnace has high accuracy (± 0.007 °C) and stability (± 0.003 °C). The coefficients of equations (9) and (10) were derived from the results obtained with Eq. (8) by regression analysis. Furthermore, the estimated values obtained with Eqs. (8), (9), and (10) were compared to the conventional equation (3). ASTM E1965-98^7^ specifies that the accuracy of IRT for use as a skin thermometer must be < 0.3 °C and IEC 80601-2-59^21^ specifies that the stability of IRT for use in fever screening must be < 0.1 °C. Therefore, we defined high accuracy and high stability as MAE < 0.3 °C and standard deviation < 0.1 °C, respectively, where MAE is defined as the mean absolute error between the values estimated by Eqs. (3), (8), (9), and (10) and the actual temperature. Accuracy was evaluated as the percentage of the number of conditions within ± 0.3 °C of the set temperature of BB-1. Using the average of 72 conditions (with three cameras, each camera was investigated under 24 conditions), the accuracy of each equation was evaluated by the *χ*^2^ test. A two-tailed *P* < 0.05 was taken to indicate statistical significance.

## Results

The dataset in this article presents the MAE calculated using Eqs. (3), (8), (9), and (10) (Appendix Table S1-S3). The MAE of IRT-2 and IRT-3 were high with the conventional equation (3). Using Eq. (3), the MAE of IRT-3 exceeded 3 °C in some cases, and the MAE values were different for IRT-1 and IRT-2 even with the same specifications.

**Figure 5:**
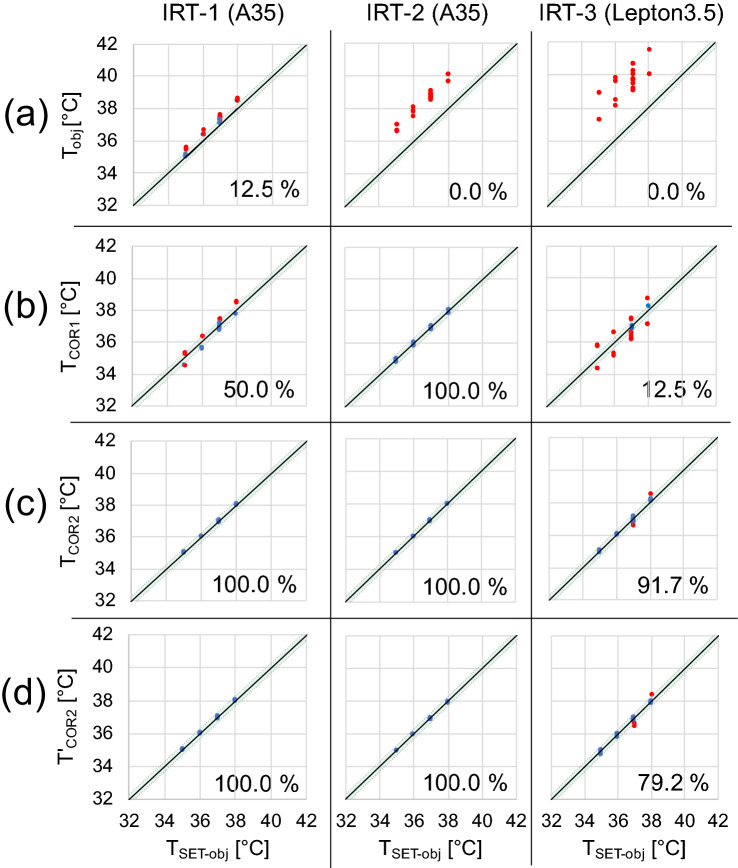
Accuracy of each correction equation and the proportion within ± 0.3 °C of the ideal temperature.

The differences between each condition and MAE for each camera are shown in Fig. 5. The blackbody furnace (*T*_SET-obj_) was set to temperatures of 35, 36, 37, and 38 °C. The solid line indicates the complete match with the ideal value and the two dashed lines indicate the range ± 0.3 °C. Blue points indicate those within ± 0.3 °C accuracy, while red points indicate those outside ± 0.3 °C. Numbers on the lower right indicate the proportion within ± 0.3 °C of the ideal temperature. The top row “(a)” was calculated using equation (3) as *T*_obj_, the second row “(b)” was calculated using equation (8) as *T*_COR1_, the third row “(c)” was calculated using equation (9) as *T*_COR2_, and the bottom row “(d)” was calculated using equation (10) as *T′*_COR2_.

The distances were limited as IRT was installed inside the incubator, and there were only small differences in the values of *τ* under each condition (*τ* range: 0.9920–0.9948).

The accuracies of the cameras are shown in Fig. 5. Using the conventional Eq. (3), *T*_obj_ tended to be higher than the ideal value (Fig. 5(a)). The standard deviation was still high despite using a relatively accurate camera, such as IRT-1 (Appendix Table S1). Equation (8) was shown to have significantly higher accuracy than Eq. (3) (54.2% vs. 4.2%, respectively; *χ*^2^ = 43.6, *P* < 0.0001) (Figs. 5(a), (b)). However, the error was affected by the measurement conditions, and values of *T*_COR1_ fluctuated depending on the measurement conditions even with BB_obj_ set to the same temperature. In addition, the error varied between cameras. Therefore, accurate values were estimated using Eq. (9) as it can offset the variation in measurement conditions and had significantly higher accuracy than Eq. (8) (97.2 vs. 54.2%, respectively; *χ*^2^ = 36.3, *P* < 0.0001) (Fig. 5(c)).

Furthermore, Eq. (10) had significantly higher accuracy than Eq. (3) (93.1 vs. 4.2%, respectively; *χ*^2^ = 113.9, *P* < 0.0001) (Fig. 5(d)), and there was no significant difference in accuracy between Eqs. (9) and (10) (*χ*^2^ = 1.4, *P* = 0.25).

## Discussion

Previous studies have measured the body temperature of neonates inside the incubator by IRT 2.^1, 2, 22, 23, 29^ In these studies, the temperature measured by IRT was compared to that determined with a thermistor as the contact temperature. However, in premature infants, the contact measurement method is also inaccurate due to characteristics of the premature skin.^22, 35^ Furthermore, previous studies on the effectiveness and accuracy of IRT applied to newborn infants were mostly conducted with the IRT placed outside the incubator, and there have been no reports on the accuracy of IRT installed inside the incubator. Therefore, we assessed the measurement accuracy of the IRT installed inside an incubator using a blackbody furnace.

The conventional Eq. (3) eliminates the external error factors, but there were conditions under which the error exceeded 10% (IRT-3, *T*_SET-incu_: 39°C, *T*_SET-obj_: 38°C, *RH*_SET-incu_: 80%) (Appendix Table S3). Equation (8) can also eliminate internal error factors because it uses the same FOV temperature differences. However, the differences between individual cameras as well as the differences in temperature between BB_obj_ and internal environment of the incubator also had an influence. Therefore, Eq. (9) used regression analysis and determined the coefficients of each camera, which resulted in improved accuracy. Moreover, Eq. (10) showed high accuracy even with reduction of the conditions from 24 to only 2.

**Figure 6:**
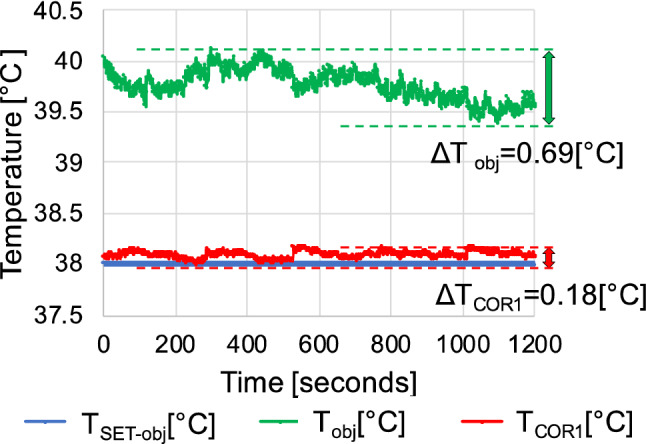
Comparison of *T*_obj_ and *T*_COR1_ over time with the corrected data using the conventional equation (3): *T*_obj_, and using equation (8): *T*_COR1_. (IRT-2, settings *T*_SET-incu_: 30°C, *T*_SET-obj_: 38°C, *RH*_SET-incu_: 90%)

Figure 6 shows that the values obtained with equation (3) were inaccurate regardless of the calibration phase using a mechanical shutter. Abbas *et al.*^1^ limited the recording time in their study due to the IRT recalibration phases. In this study, however, the errors were large even with limitation of the measurement phases. In these cases, standard two-point calibrations are required. However, the influences of temperature changes, not only of the neonate but also of the conditions inside the incubator, must be taken into consideration when applying IRT in an incubator. Therefore, frequent calibrations are required whenever the settings of the incubator are changed.^10^

Furthermore, even when using the same equipment, MAE differs regardless of the noise equivalent temperature difference (NETD) and signal transfer function (SiTF). Minkina and Dudzik^25^ reported that the accuracy of IRT is dependent on calibration by the manufacturer. In addition, Cao and Tisse^10^ and Riou *et al.*^33^ reported that outputs were different between individual detectors even when using the same equipment. In fact, the accuracy differed between detectors even using the same equipment in the present study (Fig. 5(a)). Therefore, it is necessary for manufacturers to adjust the individual equipment because most types of IRT have a wide operating range. Moreover, even if it were possible to calibrate for a narrow operating range, there would still be issues associated with thermal drift. Riou *et al.*^33^ reported that improvement of the accuracy of thermal infrared cameras required correction for thermal drift. Therefore, we adopted the relative temperature of the IRT in the present study based on the concept that all points had identical parameters as the measurements were obtained with the same detector.^25^

Our method achieved high accuracy. The conventional equation (3) showed fluctuation over time of 0.69 °C, while this was decreased to 0.18 °C with the use of Eq. (8) (Fig. 6). However, appropriate corrections could be made in some cases, but this was not possible in other cases. Even using equation (8), the influence of the incubator settings could not be eliminated (Fig. 5(b)), and there were differences in estimated values even when using the same blackbody furnace settings. Both Eqs. (3) and (8) incorporated the environmental temperature, but its influence could not be entirely eliminated. Furthermore, the mechanical shutter calibration process depends on information regarding temperature inside the housing of the IRT, and was also not sufficient to eliminate the influence of the environment. Therefore, adjustments were made by regression analysis. Equation (9) was affected by the temperature difference between the incubator settings and the object to be measured, as well as by the differences between cameras. Therefore, each camera was adjusted by regression analysis, which made it possible to measure temperatures with high accuracy at all incubator settings (Fig. 5(c)).

This calibration method takes both the measurement environment temperature and the temperature range of the object to be measured into consideration. In contrast to existing multipoint calibration methods that only consider the temperature of the object to be measured, adjustments can be made with the temperature of the incubator and the temperature range of the object to be measured. Furthermore, multipoint calibration cannot be performed with a neonate in the incubator as it requires the use of a dangerous heat source. However, a calibration source is required to deal with thermal drift.

In this study, the regression equation was initially obtained using 24 conditions for each camera, and we then confirmed that the number of conditions could be reduced to only two. Therefore, analysis of the results of these two conditions and determination of the coefficient beforehand will allow accurate measurements in other situations. However, there were differences in accuracy between IRT-1, IRT-2, and IRT-3. All conditions of IRT-1 and IRT-2 had accuracies ± 0.1 °C, whereas 20.8% of IRT-3 conditions were outside of ± 0.3°C even when using Eq. (9). Although the accuracy was the same for both A35 and Lepton 3.5, we still feel that there are limitations when using the smaller power-saving model (Table 2).

As shown in the study dataset, the standard deviation of MAE in most conditions exceeded 0.1 °C even when using the conventional Eq. (3), while Eqs. (9) and (10) achieved MAE standard deviation < 0.1 °C except for one condition (Appendix Table S1–S3), thus satisfying the requirements of IEC 80601-2-59.^21^

The high accuracy of thermography with this method is very important for the clinical use of thermography, but it is also important to capture the contours of the neonate in clinical practice. In other words, a method automatically tracking the defined ROI (region of interest) is needed in monitoring the body temperature of neonates.^3^ The neonatal body surface is small and sometimes intertwined with medical cables. Therefore, inaccurate detection of ROI leads to misanalysis of body temperature. Hence, we reported a method for the segmentation of thermal images that enables continuous non-invasive monitoring of the body temperature distribution over the whole body of neonates.^6^ The newly established equation (10) will give new evidence in this field combined with this segmentation method.

In conclusion, the Eq. (10) significantly improved the accuracy and stability of temperature measurements of subjects placed in an incubator. This study will facilitate the development of novel means of administrating neonatal body temperature.

## Supplementary Information

Below is the link to the electronic supplementary material.

Supplementary file1 (PDF 102 kb)

## Abbreviations

FOV: Field of view
NEC: Neonatal necrotizing enterocolitis
NIRT: Neonatal infrared thermography
FPA: Focal plane array
NUC: Non-uniformity correction
ETRS: External temperature reference source
NETD: Noise equivalent temperature difference
SiTF: Signal transfer function
