# Supplementary material for: Infrared Thermography with High Accuracy in a Neonatal Incubator
Source: Ann Biomed Eng. 2022 Mar 2;50(5):529–39. doi: 10.1007/s10439-022-02937-w (PMC8890465; doi:10.1007/s10439-022-02937-w)
Supplement: Supplementary file 1 — Supplementary file1 (PDF 102 kb) [file 10439_2022_2937_MOESM1_ESM.pdf]

APPENDIX

Infrared Thermography with High Accuracy in a Neonatal Incubator

TABLE S1. Accuracy of each correction equation of IRT-1 (A35)

Settings			Results			
$T_{SET\text{-}incu}$	$T_{SET\text{-}obj}$	$RH_{SET\text{-}incu}$	MAE(T_{obj}) ± SD	MAE(T_{COR1}) ± SD	MAE(T_{COR2}) ± SD	MAE(T'_{COR2}) ± SD
30	35	50	0.4744 ± 0.12	0.2857 ± 0.06	0.0470 ± 0.03	0.0467 ± 0.03
30	36	50	0.3826 ± 0.08	0.3870 ± 0.07	0.0500 ± 0.03	0.0503 ± 0.04
30	37	50	0.5163 ± 0.08	0.4749 ± 0.06	0.0499 ± 0.03	0.0508 ± 0.03
30	38	50	0.6837 ± 0.18	0.5355 ± 0.07	0.0512 ± 0.03	0.0499 ± 0.03
30	35	90	0.6144 ± 0.10	0.3266 ± 0.05	0.0379 ± 0.03	0.0393 ± 0.04
30	36	90	0.6741 ± 0.13	0.4066 ± 0.05	0.0462 ± 0.03	0.0490 ± 0.03
30	37	90	0.7455 ± 0.12	0.4811 ± 0.05	0.0400 ± 0.03	0.0436 ± 0.03
30	38	90	0.7429 ± 0.13	0.5750 ± 0.06	0.0515 ± 0.04	0.0566 ± 0.04
31	37	50	0.3952 ± 0.13	0.4177 ± 0.05	0.0444 ± 0.03	0.0475 ± 0.03
33	37	50	0.5703 ± 0.09	0.2093 ± 0.05	0.0430 ± 0.03	0.0423 ± 0.03
35	37	50	0.5587 ± 0.15	0.0456 ± 0.03	0.0625 ± 0.04	0.0644 ± 0.04
35	37	60	0.5814 ± 0.11	0.0380 ± 0.03	0.0679 ± 0.04	0.0697 ± 0.04
35	37	70	0.4551 ± 0.14	0.0371 ± 0.02	0.0654 ± 0.04	0.0676 ± 0.04
35	37	80	0.5827 ± 0.11	0.0513 ± 0.03	0.0812 ± 0.04	0.0839 ± 0.04
37	37	50	0.3214 ± 0.21	0.1082 ± 0.03	0.0245 ± 0.02	0.0225 ± 0.02
37	37	90	0.4285 ± 0.10	0.0972 ± 0.03	0.0448 ± 0.03	0.0384 ± 0.03
39	35	50	0.1135 ± 0.06	0.4335 ± 0.03	0.0280 ± 0.02	0.0252 ± 0.02
39	36	50	0.4188 ± 0.10	0.3625 ± 0.04	0.0285 ± 0.02	0.0267 ± 0.02
39	37	50	0.1729 ± 0.12	0.2464 ± 0.05	0.0417 ± 0.03	0.0391 ± 0.02
39	38	50	0.5558 ± 0.16	0.1954 ± 0.03	0.0272 ± 0.02	0.0265 ± 0.02
39	35	80	0.1794 ± 0.10	0.4644 ± 0.04	0.0292 ± 0.02	0.0294 ± 0.02
39	36	80	0.3947 ± 0.12	0.4014 ± 0.03	0.0265 ± 0.02	0.0293 ± 0.02
39	37	80	0.4614 ± 0.15	0.2785 ± 0.03	0.0349 ± 0.02	0.0296 ± 0.02
39	38	80	0.5745 ± 0.09	0.2249 ± 0.03	0.0237 ± 0.02	0.0257 ± 0.02

TABLE S2. Accuracy of each correction equation of IRT-2 (A35)

Settings			Results			
$T_{SET\text{-}incu}$	$T_{SET\text{-}obj}$	$RH_{SET\text{-}incu}$	MAE(T_{obj}) ± SD	MAE(T_{COR1}) ± SD	MAE(T_{COR2}) ± SD	MAE(T'_{COR2}) ± SD
30	35	50	1.7194 ± 0.11	0.0356 ± 0.02	0.0302 ± 0.02	0.0253 ± 0.02

30	36	50	1.9119	±	0.15	0.0376	±	0.02	0.0220	±	0.02	0.0249	±	0.02
30	37	50	1.5956	±	0.14	0.0557	±	0.03	0.0234	±	0.02	0.0254	±	0.02
30	38	50	1.7606	±	0.18	0.0863	±	0.03	0.0324	±	0.02	0.0255	±	0.02
30	35	90	1.6060	±	0.13	0.0320	±	0.02	0.0314	±	0.02	0.0365	±	0.03
30	36	90	1.6084	±	0.13	0.0322	±	0.03	0.0258	±	0.02	0.0301	±	0.02
30	37	90	1.7163	±	0.10	0.0590	±	0.03	0.0279	±	0.02	0.0256	±	0.02
30	38	90	1.7469	±	0.15	0.0831	±	0.03	0.0299	±	0.02	0.0255	±	0.02
31	37	50	1.8682	±	0.15	0.0476	±	0.02	0.0274	±	0.02	0.0214	±	0.02
33	37	50	1.7391	±	0.12	0.0421	±	0.03	0.0351	±	0.03	0.0535	±	0.03
35	37	50	1.9107	±	0.22	0.0713	±	0.04	0.0292	±	0.03	0.0434	±	0.03
35	37	60	1.7803	±	0.12	0.0717	±	0.03	0.0300	±	0.02	0.0476	±	0.03
35	37	70	2.0209	±	0.16	0.0862	±	0.03	0.0374	±	0.03	0.0588	±	0.03
35	37	80	1.9297	±	0.17	0.0913	±	0.02	0.0392	±	0.02	0.0636	±	0.02
37	37	50	2.0135	±	0.13	0.1076	±	0.02	0.0193	±	0.02	0.0378	±	0.02
37	37	90	1.8506	±	0.14	0.0985	±	0.02	0.0135	±	0.01	0.0263	±	0.02
39	35	50	2.0283	±	0.12	0.1440	±	0.02	0.0314	±	0.01	0.0119	±	0.01
39	36	50	2.0122	±	0.18	0.1267	±	0.03	0.0331	±	0.02	0.0206	±	0.01
39	37	50	2.0234	±	0.14	0.1775	±	0.02	0.0562	±	0.02	0.0848	±	0.02
39	38	50	2.2300	±	0.18	0.1141	±	0.02	0.0173	±	0.01	0.0259	±	0.02
39	35	80	2.0402	±	0.21	0.1605	±	0.02	0.0248	±	0.02	0.0182	±	0.01
39	36	80	2.1730	±	0.12	0.1519	±	0.02	0.0179	±	0.01	0.0197	±	0.01
39	37	80	2.1673	±	0.13	0.1319	±	0.02	0.0197	±	0.02	0.0237	±	0.01
39	38	80	2.2041	±	0.11	0.1376	±	0.02	0.0173	±	0.01	0.0402	±	0.02

TABLE S3. Accuracy of each correction equation of IRT-3 (Lepton3.5)

Settings			Results											
$T_{SET-incu}$	$T_{SET-obj}$	$RH_{SET-incu}$	MAE(T_{obj}) ± SD			MAE(T_{COR1}) ± SD			MAE(T_{COR2}) ± SD			MAE(T'_{COR2}) ± SD		
30	35	50	2.3758	±	0.44	0.6374	±	0.06	0.0550	±	0.04	0.1383	±	0.06
30	36	50	2.2154	±	0.44	0.8155	±	0.06	0.0561	±	0.04	0.1885	±	0.06
30	37	50	2.1769	±	0.42	0.8467	±	0.05	0.0962	±	0.06	0.0915	±	0.05
30	38	50	2.0993	±	0.40	0.8929	±	0.05	0.1970	±	0.05	0.0428	±	0.03
30	35	90	2.3663	±	0.46	0.6807	±	0.07	0.1136	±	0.07	0.2558	±	0.07
30	36	90	2.5909	±	0.70	0.7163	±	0.06	0.0648	±	0.04	0.1308	±	0.06
30	37	90	2.1331	±	0.46	0.8217	±	0.05	0.0887	±	0.06	0.0974	±	0.06
30	38	90	2.1136	±	0.43	0.9181	±	0.05	0.0968	±	0.06	0.0984	±	0.05
31	37	50	2.3152	±	0.45	0.7275	±	0.05	0.0884	±	0.05	0.0884	±	0.05
33	37	50	2.5675	±	0.43	0.5907	±	0.05	0.0888	±	0.05	0.2264	±	0.06
35	37	50	2.8040	±	0.48	0.3984	±	0.09	0.2502	±	0.10	0.3570	±	0.10

35	37	60	2.8236	±	0.49	0.3988	±	0.08	0.2428	±	0.09	0.3509	±	0.09
35	37	70	2.7871	±	0.46	0.4242	±	0.08	0.2755	±	0.09	0.3830	±	0.09
35	37	80	2.8179	±	0.49	0.4920	±	0.08	0.3611	±	0.10	0.4670	±	0.10
37	37	50	3.0992	±	0.48	0.0787	±	0.04	0.1388	±	0.04	0.2266	±	0.04
37	37	90	3.3683	±	0.40	0.0778	±	0.03	0.0949	±	0.04	0.1720	±	0.04
39	35	50	3.9683	±	0.39	0.7070	±	0.07	0.0748	±	0.04	0.0659	±	0.04
39	36	50	3.7083	±	0.42	0.6048	±	0.07	0.0961	±	0.05	0.0715	±	0.04
39	37	50	3.7368	±	0.41	0.4391	±	0.07	0.0919	±	0.04	0.0666	±	0.04
39	38	50	3.6039	±	0.38	0.2700	±	0.04	0.0534	±	0.03	0.0446	±	0.04
39	35	80	3.9927	±	0.37	0.7859	±	0.07	0.0991	±	0.06	0.0846	±	0.05
39	36	80	3.9136	±	0.42	0.6088	±	0.07	0.0938	±	0.05	0.0754	±	0.04
39	37	80	3.8000	±	0.35	0.4777	±	0.05	0.0930	±	0.05	0.0586	±	0.03
39	38	80	4.1126	±	0.38	0.7347	±	0.07	0.5296	±	0.09	0.4555	±	0.09
